# Are agricultural commodity production systems at risk from local biodiversity loss?

**DOI:** 10.1098/rsbl.2024.0283

**Published:** 2024-09-18

**Authors:** Calum Maney, Marieke Sassen, Ken E. Giller

**Affiliations:** ^1^ Plant Production Systems, Wageningen University, P.O. Box 430, Wageningen 6700, The Netherlands; ^2^ UN Environment Programme World Conservation Monitoring Centre (UNEP-WCMC), 219 Huntingdon Road, Cambridge CB3 0DL, UK

**Keywords:** ecosystem services, agricultural biodiversity, agricultural commodities, pollination, pest control, soil health

## Abstract

Compelling evidence for feedbacks between commodity crop production systems and local ecosystems has led to predictions that biodiversity loss could threaten food security. However, for this to happen agricultural production systems must both impact and depend on the same components of biodiversity. Here, we review the evidence for and against the simultaneous impacts and dependencies of eight important commodity crops on biodiversity. We evaluate the risk that pollination, pest control or biodiversity-mediated soil health maintenance services are at risk from local biodiversity loss. We find that for key species groups such as ants, bees and birds, the production of commodities including coffee, cocoa and soya bean is indeed likely to be at risk from local biodiversity loss. However, we also identify several combinations of commodity, ecosystem service and component of biodiversity that are unlikely to lead to reinforcing feedbacks and lose–lose outcomes for biodiversity and agriculture. Furthermore, there are significant gaps in the evidence both for and against a mutualism between biodiversity and agricultural commodity production, highlighting the need for more evaluation of the importance of specific biodiversity groups to agricultural systems globally.

## 1. Commodity crop production intersects with the threatened biodiversity that underpins ecosystem services

Much of the world’s agricultural land lies within biodiversity-rich areas and is used to produce commodities ‘for export’ at the expense of local biodiversity [1,2]. The expansion and intensification of agricultural production have been rapid and are among the most important drivers of global biodiversity loss [3]. At the same time, biodiversity in agricultural landscapes underpins several ecosystem functions, such as wild pollinator activity and the cycling of soil nutrients [4,5]. These functions contribute to the stability of agricultural systems [6] and their resilience to fluctuations in environmental conditions [7,8]. Human-mediated impacts on ecosystem services are also increasing: indicators designed to measure pollination, climate, freshwater quality and quantity, the regulation of biological processes, and the provision of food and materials all show considerable declines since 1970 [9].

**Figure 1.**
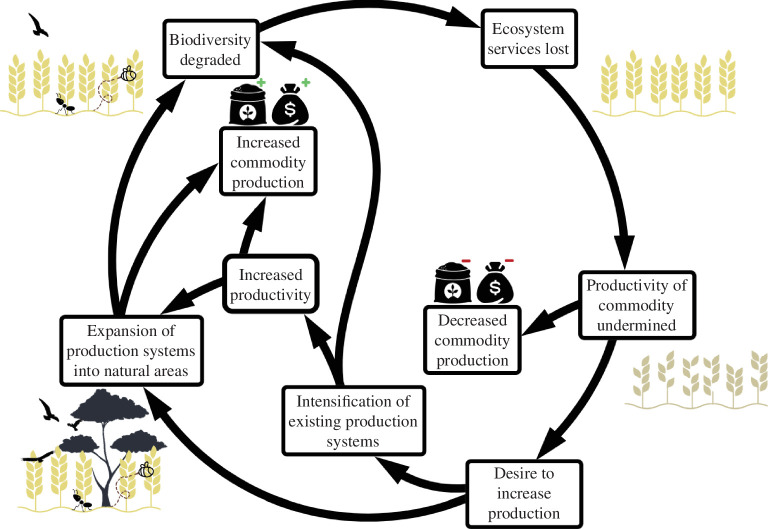
Ecosystem service feedbacks may drive a reinforcing feedback loop with negative outcomes if production is dependent on ecosystem services. Arrows represent hypothesized causal relationships.

Observable relationships between agricultural biodiversity and desirable outcomes have led to the idea of a ‘biodiversity–production mutualism’ in agricultural landscapes. This theory suggests that the functions associated with biodiversity contribute to agricultural production [10]. Accordingly, if agricultural expansion and intensification exceed a sustainable limit, ecosystem services will be degraded and crop yields reduced through the loss of functional groups of biodiversity [11–13]. For example, increasing demand for commodities that depend on pollination services makes the delivery of pollination services more important to global production landscapes [11]. At the same time, increasing land-use intensity and the expansion of cropland are associated with falling richness and abundance of insect pollinators [14]. Systems that impact ecosystem services such as pollination too heavily could become caught in an ‘intensification trap’, whereby they become increasingly dependent on manufactured inputs to replace lost ecosystem services, reducing the profitability of the system in a lose–lose situation for biodiversity and agriculture [15,16] (figure 1).

At the global scale, simultaneous increases in crop system impacts and dependencies on biodiversity could lead to a future where the production of key commodity crops is jeopardized [13]. Conversely, the ‘interdependence’ of agriculture and biodiversity means that nature-based solutions could be valuable to support biodiversity and consequently, food production. Utilizing nature-based solutions to capitalize on the biodiversity–production mutualism may even reduce the need for continued expansion of agriculture into natural areas [17]. Interdependencies are well-known for some species groups such as birds, where context-specific examples of impacts and dependencies are well-described [18,19]. However, the biodiversity–production mutualism is a broad concept, which lacks detailed causal evaluation, and needs contextualization.

## 2. Projecting the impacts of biodiversity decline on agriculture may overlook context-specificity

Evidence for a biodiversity–production mutualism and the risk of ‘intensification traps’ has fuelled powerful statements about the value of biodiversity to agriculture [20,21]. This evidence also informs recent global analyses of the risks that declining biodiversity may pose to global agriculture and food security [11,13,22,23]. Global-scale projections of the future of biodiversity and agriculture that incorporate interdependencies between the two often predict large shocks to food systems driven by biodiversity loss [13,15]. At the global level, these findings are important: they highlight a pressing concern that warrants policy responses commensurate with the threat posed to people and nature. However, the biodiversity–agriculture interdependencies underpinning these findings are based on, often broad, theoretical assumptions [16,24]. Dependency relationships in particular tend to be simplified at least to crop level; for pollination, this often means using the classification of pollination dependency from Klein *et al*. [25] alongside estimates of habitat proximity (e.g. [23]) or an assumption of linear decreases in yield proportional to crop dependence on pollination (e.g. [13]). Yet, when considering practical options to improve agronomic and ecological outcomes in individual cropping systems, it is important to consider in context the specifics of which biodiversity is important, and the extent to which it is threatened by agriculture [26].

The relationship between landscape-level natural habitat, functional group biodiversity, ecosystem service provision and crop production has been summarized quantitively across many crops and components of biodiversity. Overall patterns indicate evidence of interdependence between agriculture and biodiversity [27]. However, analyses to estimate interdependence in individual cropping systems or for specific components of biodiversity are limited by the availability of data, and patterns at the global level may not hold in specific contexts.

Realistically, in any given instance, only a small proportion of species are likely to contribute directly to ecosystem services and yield [28]. These are also often the most abundant species within a given functional group [29], which weakens the overall argument for biodiversity–productivity co-benefits. While there is strong evidence that biodiversity–production mutualisms exist, they are not ubiquitous and need to be established in each case [30,31]. Depending on the specific circumstances, priorities to protect ecosystem service provision and actions to protect biodiversity may not always align [28,31]. These circumstances can be related to many properties of the production system, including geographic region, farm scale and cropping system [32–34]. At best, the argument to increase *all* biodiversity within agricultural systems may be inefficient in supporting production. At worst, holding biodiverse agroecosystems as an ideal could risk threatening food security and local livelihoods. Further, farmers can reduce their dependence on natural systems by introducing non-natural substitutes and enhancements. For example, by using bee hives, farmers may reduce the dependence they would otherwise have on nearby forests to provide pollinators [35]. Smallholder or ‘family’ farmers are often particularly dependent on ecosystem services for crop production as they often have fewer resources to invest in external inputs, especially in the tropics (e.g. [36]). They produce 30% of global food supply [37], highlighting the importance of ensuring tipping points of biodiversity degradation are not crossed.

Crop failure can mean disaster for farmers, so agronomic and ecological research must be thorough and nuanced when evaluating biodiversity as a tool to sustain productivity. If trust and enthusiasm for protecting local biodiversity to prevent food system collapses is to be garnered within agricultural communities, the risk of interdependencies that lead to productivity declines must be established in real-world commodity production systems. Of course, there are many reasons to conserve biodiversity beyond a recognition of its contributions to people—moral, ethical and biocultural values also guide human interactions with nature [38]. Where such other reasons to conserve a component of biodiversity dominate but are not shared by farming communities, incentives and/or compensation for any trade-offs with farming will need to be found.

## 3. Investigating interdependencies with a case study approach

The goal of this review is to establish, for a range of crops, whether a reinforcing feedback between biodiversity loss and low productivity is likely to exist. We began by combining existing evidence from agronomic and ecological studies. Due to the varied nature of the evidence collected, it was impractical to conduct a quantitative meta-analysis such as has been done for individual services, crops or biodiversity features globally [27,39]. Instead, we opt for a case study and narrative approach, focusing on the findings of specific studies, to identify the balance of evidence either supporting or contradicting the two hypotheses. To do this, we reviewed evidence of how biodiversity supports productivity, comparing it to the evidence that those production systems put the same biodiversity at risk. We focus on eight economically and ecologically significant commodity crops that are predominantly exported and are known to grow in areas of high biodiversity [40,41]. Such a selection limits the findings of our review to a specific set of socio-economic and geographic conditions but should more reliably capture systems where there are material impacts of agriculture of biodiversity and vice-versa. These were cocoa (*Theobroma cacao* L.), coffee (*Coffea arabica* L.; also *C. canephora* P. and others), cotton (*Gossypium hirsutum* L.), oil palm (*Elaeis guineensis* Jacq.), rubber (*Hevea brasiliensis* (Willd. ex A. Juss.) Müll. Arg.), soya bean (*Glycine max* (L.) Merr), sugar cane (*Saccharum officinarum* L.) and tea (*Camellia sinensis* (L.) Kuntze).

Our review is targeted at evaluating the evidence for and against two hypotheses, both of which must be true for reinforcing feedbacks between biodiversity loss and low productivity to occur. The first is that, at the local level, the productivity of commodity cropping systems depends upon specific components of biodiversity. The second is that these same components of biodiversity are impacted negatively by the expansion and intensification of specific cropping systems. Following literature searches using the Web of Science tool (https://www.webofscience.com/) and constructed search terms (electronic supplementary material, table S1), we identified the components of biodiversity implicated in delivering ecosystem services. For each component, we reviewed the evidence that cropping systems benefit from biodiversity, focusing on studies which measured both a component of biodiversity and a measure of productivity. We then reviewed the evidence on the impacts of the cropping system impacting each component. This limited our review to components of biodiversity that are already known or assumed to be useful to production systems.

**Figure 2.**
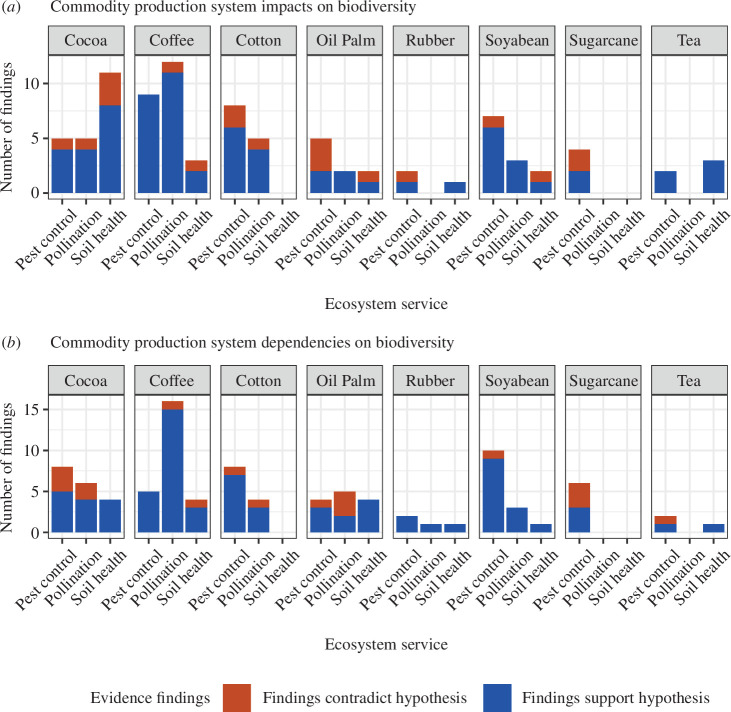
The distribution of evidence collected in the review, arranged by crop and ecosystem service. (*a*) Evidence collected on the hypothesis of dependence. (*b*) Evidence collected on the hypothesis of impacts. Fill colour represents the balance between evidence broadly supporting hypotheses, where red (the top colour) represents the number of findings not supporting the hypothesis or with only context-specific support for it.

**Figure 3.**
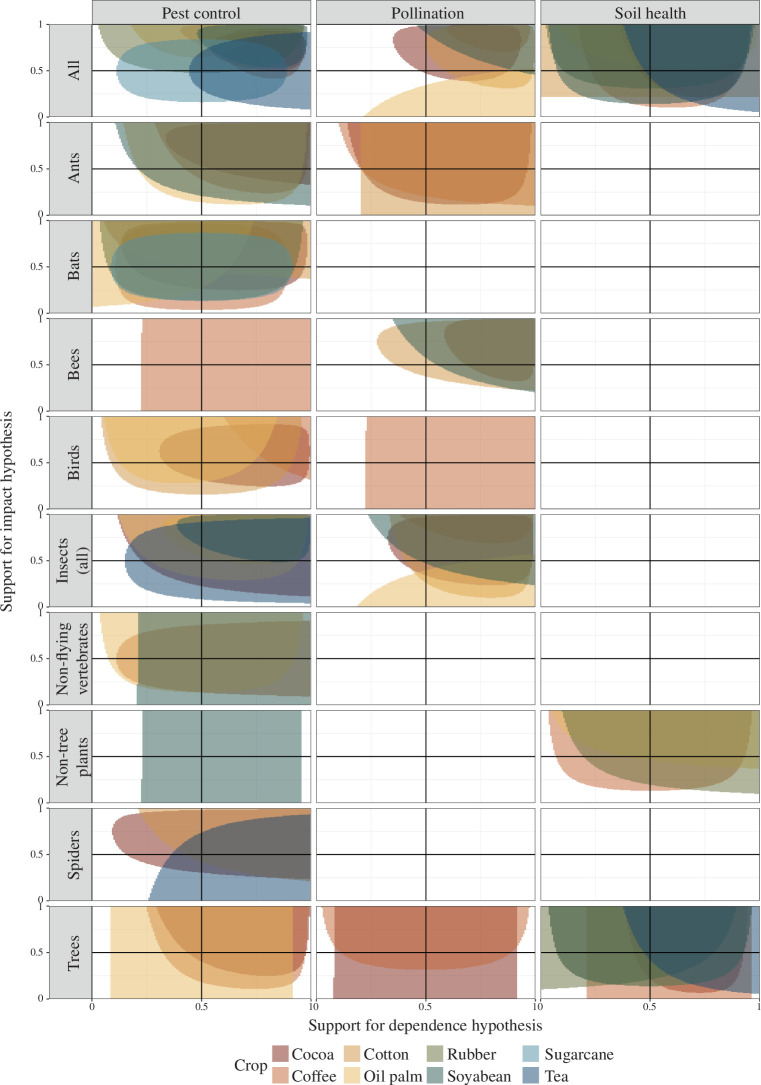
The evidence bases for ecological impacts and agronomic dependencies of commodity crop production systems on biodiversity. Shaded areas mostly in the top-right quadrant belong to combinations of crop, ecosystem service and biodiversity component, where evidence is likely to support the hypothesis that interdependencies, feedback loops and ‘intensification traps’ could emerge due to crop-induced biodiversity change. The shaded regions represent the 95% percentile mass of the joint posterior distribution of findings’ support for the impact and dependence hypotheses in the reviewed evidence. This therefore represents the likely outcome of seeking both evidence for dependence and evidence for impacts of a given cropping system via one of the three ecosystem services. A complete breakdown of the results by crop is also available for clarity (electronic supplementary material, figure S2).

Our initial searches identified a total of 159 scientific papers containing findings about the impacts and dependencies of commodity production systems on biodiversity. This limited number of findings was surprising, though perhaps less so when the specific requirements for evidence on ‘dependency’ are considered. There are only a small proportion of published articles concerning biodiversity and ecosystem services that directly relate the diversity of a component of biodiversity, through the provision of an ecosystem service, to productivity in crops. For instance, the CropPol database, the largest open database on biodiversity–crop pollinator interactions, covers 202 studies over 32 crops—a much larger range than the scope of this study [42]. Thus, we were satisfied a genuine data gap has been identified. Our review covered nine components of biodiversity, some of which were nested, such as ‘ants’ within ‘insects’. Our follow-up searches for impacts and dependencies of production systems on the components of biodiversity uncovered 46 further sources, including for derivative and prior research linked to sources in the original search, and those in languages other than English. In total, our review covered eight commodity crops, three ecosystem service categories and nine main components of biodiversity. The total number of ‘findings’ was 151 for dependencies of crop systems on biodiversity, and 163 for impacts of crop systems on biodiversity. We summarized these in both a narrative format (electronic supplementary material, table S2) and figures (figures 2 and 3).

To illustrate how these findings translate into the likelihood of observing effects of interdependencies in the eight target cropping systems, we identified findings from the context-specific evidence base simply as supporting (*Y*), mixed (*Y* and *N*) or contradictory (*N*) to the dependency impact hypotheses. We then summarized the evidence base in a simple Bayesian framework [43], with a prior assumption that finding evidence to support or contradict the two hypotheses was equally likely:


$$Y_{dependence}\;∼\;Binomial\left(n_{dependence},p_{dependence}\right)$$

$$p_{dependence}\;∼\;Uniform\left(0,1\right)$$

$$Y_{impact}\;∼\;Binomial\left(n_{impact},\;p_{impact}\right)$$

$$p_{impact}\;∼\;Uniform\left(0,1\right)$$

$$R=Y_{dependence}\;\cdot \;Y_{impact},$$

where *n* represents the number of findings related to each of the two hypotheses that were identified for each combination of crop, service and component of biodiversity; the *Y* variables represent the number of those studies supporting the hypotheses and overall risk, *R*, represents the probability that two pieces of evidence, one concerning each hypothesis, will reflect impacts and dependencies of a cropping system on a component of biodiversity.

## 4. The evidence base for impacts and dependencies is distributed differently among biodiversity groups

As reflected in the combined evidence for impacts and dependencies of agricultural systems on wild biodiversity [27], the findings of this review show consistent evidence both for agriculture degrading the diversity of potentially beneficial species groups and for the measurable benefits of those species groups to productivity. However, the evidence base for impacts and dependencies is distributed differently among biodiversity groups. When results are separated by their relevant component of biodiversity, evidence concerning some species groups is weaker or may even indicate a lack of dependence or impact (figure 3; electronic supplementary material, figure S2).

Rather than reiterating the findings of each study contributing to the results of this review (figure 3; electronic supplementary material, table S2), here we discuss illustrative case studies, apparent data gaps and departures from the overall trend that interdependencies between agriculture and biodiversity are present in the reviewed commodity production systems.

The evidence base substantiates pollination service interdependencies across most investigated components of biodiversity. This reinforces the value of broadly applied models of this ecosystem service, which typically focus on bee species [44]. While the evidence for interdependence in the case of other pollinator groups was more mixed, it was still mostly supported. The evidence for interdependencies was strongest in coffee and cotton, though benefits have been found to depend on important interactions between pollinators and other insects.

Oil palm systems present a well-known departure from typical pollination interdependencies. Oil palm pollination depends on the oil palm weevil *Rhynchophorus ferrugineus*. This species lives within the palm itself and is not known to be affected by the habitat degradation typically associated with oil palm production. Yet periods of heavy rainfall and nematode infections have been associated with pollinator declines and low yield in oil palm [45]. More research is warranted on interdependencies linked to pollination in some of our crops. For example, cocoa is highly dependent on pollination [25], cocoa yields are low in most production systems, especially in West Africa and hand pollination is known to increase yields [46–48]. The abundance of *Diptera* in cocoa plantations increased with increasing shade and shade complexity in one study [49]. Yet other practices with narrower biodiversity co-benefits, such as adding rotting banana pseudostems to farms, can also benefit pollination [50]. The evidence suggests that enhanced pollination services would benefit cocoa production, but the key target pollinator species and the best ways to promote them remain uncertain.

There are key discrepancies in some findings related to biodiversity dependencies, for instance, in soya bean pollination. One recent article argues that current systems ignore the importance of this service to the crop, and its potential to help maintain global soya bean production while sparing land for biodiversity [17]. Indeed, different studies find that insect pollinators may contribute to soya bean productivity [44,51–55], and the design and management of soya bean production systems mediate the diversity of insect pollinators [54,56,57]. This suggests that soya bean production systems may experience feedback loops related to pollination interdependency. Yet soya bean is a self-fertilizing crop; how this can be reconciled with the observed contributions of pollinators to soya bean yields is debated [34]. Secondary mechanisms, such as the revival of relegated flowers when pollen is delivered to them, have been suggested as an explanation [52], though further research is needed to make a stronger case for biodiversity within the production system from the perspective of soya bean growers. Overall, nature-based solutions to pollination-limitation, such as on-farm habitat provision [58,59], are likely to become more important as pollinator-dependent commodity crops expand and intensify worldwide [11]. Indeed, some coffee and cotton supply chains already integrate knowledge of this interdependence in their policies and strategies. For example, Texas, the largest cotton-producing state in the USA, has a pollinator conservation plan targeting butterflies [60]. Nestlé’s ‘bees for coffee’ project [61] seeks to protect pollinators to benefit their ‘regenerative agriculture’ plans.

For pest control, known to be a more varied and context-specific ecosystem service than pollination [62], the evidence showed a more variable risk of interdependencies. Departures from generalized expectations of both impacts and dependencies on biodiversity were found for bats, birds, non-flying vertebrates and spiders across multiple cropping systems. Most of the studies on the benefits of flying predators did not consider birds and bats separately [63,64]. In the few studies that did separate effects, benefits from bats were lower [65,66], suggesting that birds may have driven most of the service provision in the mixed studies. Studies on invertebrates generally supported both the dependence and impact hypotheses [67,68], though some groups such as spiders were not studied in all systems. A recent review of the economic benefits of pest suppression by bats found that no exclosure experiment to measure the pest control benefits of bats in cotton had been carried out [39]. This may be particularly pertinent in Southeast Asia and the Amazon, which are both global centres of cotton production and bat diversity [69,70]. We did not find any study linking natural enemy diversity to rubber or sugar cane production. Yet, these commodity crops can have significant negative impacts on the diversity of the species groups implicated as natural enemies in other crop production systems [71–73], so research into their role in these systems is warranted. Conversely, while spiders were identified as a potential group around which a feedback could develop in tea, the negative impacts of tea plantations on spider diversity were found to be limited [74,75]. Non-flying mammals were not typically implicated as beneficial natural enemies (e.g. [76]), and though there was evidence for pythons as predators of the common rodent pests in oil palm, python diversity has not been linked to yield [77]. Finally, in agroforestry systems such as those used in cocoa and coffee production, we identified evidence for knock-on benefits to pest control from trees, though more evidence supporting specific species, benefits and potential trade-offs is needed to fully understand interdependencies in these systems [78,79].

Regarding biodiversity-mediated soil health services, evidence for production benefits was sparser than for other services, though generally positive towards both hypotheses. Evidence largely revolved around plant species associated with cocoa, coffee and tea production systems. Trees supporting soil health made up most of the evidence [80–82], though the structure, placement and identity of trees might be more important than tree diversity itself [83,84]. In systems such as cocoa, there are also important trade-offs with tree densities, as shade removal allows for higher cocoa planting densities, and too much shade may enhance the spread of common fungal diseases [85]. For tea, studies so far have found positive results for single-species agroforestry using alder and gingko trees [86,87]. As tea plantations are often considered detrimental to biodiversity at the site and landscape scale [71], further research into more complex mixes, and the impacts they have on biodiversity, is warranted. Identifying the best range and diversity of species to include in agroforestry systems allows for fine-tuning ecosystem service provision and the maximization of benefits [88].

Evidence for biodiversity-mediated benefits to the production of sugar cane and soya bean via soil health services was limited; some evidence on benefits from non-tree plants was found, but this was relatively sparse. Increases in soil organic carbon have been associated with incorporating woody vegetation into soya bean systems [89], but no direct connection to productivity was observed. Continuous sugar cane replanting harms both soil biodiversity and production [90]; therefore, studies into any dependence on biodiversity in sugar cane systems have the potential to reveal interdependencies.

### (a) The case of soil microbial diversity

During our search, we identified several studies that implied a role for soil microbial diversity in supporting the focal crop production systems. Most of these studies involve fertility treatments or other soil interventions where microbial diversity is also a response variable [91–94]. Studies based on interventions that increase both fertility and microbial diversity or activity cannot establish causality between them and, therefore, do not provide reliable evidence.

## 5. Takeaways and lessons for practice and policy

Broadly, the evidence examined in this study supports the notion that negative consequences can emerge through the impacts of agricultural commodity production on specific groups of local biodiversity, as reflected in recent syntheses [27,95]. Still, interdependencies between nature and agriculture are unique to crop, service and biodiversity component combinations. Even within similar combinations of crop, service and component of biodiversity, different patterns emerge. For example, while both wild bees and butterflies were implicated in delivering pollination services in coffee, the evidence base was much stronger for bees. Geographic patterns in crop impacts and dependencies further complicate relationships (e.g. [34]). Studies investigating only broad functional groups make an inadequate case for protecting groups at a finer taxonomic level that do not provide tangible benefits. Maintaining landscape-level habitat and overall ecosystem health in agricultural matrices overall seems likely to provide benefits to biodiversity with feedbacks benefitting the agroecosystem [24,27]. However, it is important for the content of both public policy and private-sector initiatives to reflect a context-specific and nuanced understanding of biodiversity–agriculture interdependencies.

Across the evidence in this review, the most consistent driver of reinforcing feedbacks and lose–lose situations for agriculture and biodiversity was related to insects, which were consistently found to both support production and be impacted by changes to farms and farming landscapes—including pesticide use [96,97]. Tree diversity was also linked to a variety of on-farm benefits as well as knock-on biodiversity and ecosystem service benefits [85,98].

Not all beneficial species are of conservation concern. Many of the useful plants identified by studies in this review are specific species, and not necessarily wild, natural or native to the regions where their benefits have been measured. This was the case for many cover crops, as well as for some trees [86,87,99].

When land-use change or land-use intensification impact biodiversity, there are ‘winners’ and ‘losers’ among groups of species: some are strongly impacted, while others thrive in modified environments [100]. In some cases, the species responsible for providing benefits to cropping systems may actually be the ‘winners’, reducing the risk of biodiversity-mediated feedbacks on agricultural production even as impacts on biodiversity at large grow [28].

Charismatic, unconventional service providers may benefit commodity production systems, as shown by a scattered but significant amount of evidence. These species, including marsupials in soya bean [101], owls in oil palm [102], and reptiles in cocoa and coffee [103,104], are not usually accounted for in large-scale analyses of biodiversity-mediated ecosystem services. However, human–wildlife conflict remains a pertinent barrier to the suitability of some of these species for promotion as nature-based solutions [105]. While charismatic species associated with pest control services may come from groups typically associated with conservation gains [106], decision-makers need to consider potential local-scale trade-offs, such as danger to people, livestock, crops [105] and property [107].

The development of effective solutions and generating trust in them requires avoiding oversimplifications. The chain of causality between interventions, biodiversity responses, ecosystem functions, ecosystem service provision and production outcomes should always be established through context-specific studies. Though interdependencies are likely in many cropping systems, it is important to avoid making projections of agricultural outcomes related directly to biodiversity loss, where evidence is currently limited or not indicative of interdependencies for the relevant species and crop combinations. Continuing to evaluate and publish evidence when biodiversity is found not to support agriculture, or found to thrive in an intensified environment, is crucial to developing and refining reliable interventions. Furthermore, this will help to establish where alternative options to enhancing ecosystem services are most appropriate for different farming systems. Smallholders in particular should have the right to ‘substitute’ for nature when their livelihoods are dependent on optimal decision-making [35].

Many of the studies included in this review were carried out in systems where some kind of nature-based solution or biodiversity-friendly practice was already implemented. This does not match the reality of many commodity crop production systems, which can be extensive and intensified [108]. Biodiversity baselines are therefore needed for many species groups and cropping systems, especially in intensive agriculture. If we are to understand the extent and importance of feedbacks in commodity production systems, we must also survey biodiversity and ecosystem services in more intensive systems and degraded landscapes to assess the extent to which biodiversity and ecosystems are also degraded. The ‘green deserts’ of large-scale, intensified agricultural commodity production systems have an as-yet-unknown potential for feedbacks between their biodiversity and productivity for most of the crops in this study. Further, there was geographic bias in the studies in this review. When the locations where evidence was found were compared to the overall growing regions of the crops, some areas had evidence focused on impacts or dependence only; other regions represented evidence gaps (electronic supplementary material, figure S1).

Policies are now in place attempting to limit the damage done to natural ecosystems by commodity production systems. The EU restoration law has specific elements addressing pollinators and farmland birds, among other important biodiversity components associated with ecosystem services in agriculture [109]. The EU anti-deforestation regulation [110] also attempts to address the demand-driven impacts of globally traded commodities. This regulation covers, among other crops, cocoa, coffee, oil palm, soya bean and rubber, for which we found evidence for reinforcing feedbacks driven by on-farm changes. If countries importing agricultural commodities wish to stop their trading activities from driving further deforestation, collapses in production driven by feedbacks must also be avoided. Furthermore, countries importing large quantities of agricultural commodities from biodiversity-rich areas need to strike a balance between protecting and restoring their own local biodiversity and protecting biodiversity in the overseas landscapes where commodities are produced [111].

Future trends in global trade will undoubtedly drive further expansion and intensification of key agricultural commodity production systems. Understanding the intricate and context-specific relationships between land-use intensity, specific components of biodiversity, ecosystem services and productivity is crucial to determining the hard limits of such intensification and expansion. Developed rigorously, and implemented well in decision-making, this understanding may be able to circumvent a scenario of biodiversity loss, service failure and unsustainable production.

## Data Availability

A list of the full collection of resources used in this analysis, and all the code needed to produce the figures in this manuscript has been made available (Supplementary Information) [112].
